# Immune thrombocytopenia after bee venom therapy: a case report

**DOI:** 10.1186/s12906-016-1091-3

**Published:** 2016-03-25

**Authors:** Mohammad Adel Abdulsalam, Bader Esmael Ebrahim, Ahmad Jasem Abdulsalam

**Affiliations:** Department of Internal Medicine, Mubarak Al-Kabeer Hospital, P.O. Box 800, Dasman, 15458 Kuwait

**Keywords:** Immune, Thrombocytopenia, Bee, Venom, Therapy, Hematology, Complication, Acupuncture, PLA2

## Abstract

**Background:**

Immune thrombocytopenia (ITP) is a hematological disorder with an isolated decrease in number of circulating platelets. Bee venom therapy (BVT) is a form of alternative medicine. It is still being practiced in the Middle East and other parts of Asia. In BVT, acupuncture points are used to inject diluted bee venom into the body. The pharmacological basis behind BVT is not fully understood. However, it has been used to treat various medical conditions such as arthritis and low back pain. On the other hand there have been a number of reported complications of BVT use such as ITP. We present a case report on ITP after BVT.

**Case presentation:**

A 61 year old lady presented with gum bleeding and ecchymosis and found to have isolated thrombocytopenia (platelet count of 9 × 10^9^/L) after receiving four direct bee sting sessions. There was no evidence of any other risk factors of ITP.

**Conclusion:**

Bee venom components and toxicity may be associated with thrombocytopenia as a complication. Further research is needed to postulate guidelines and protocol for BVT. In the meantime, monitoring of the practice of BVT should be made, with an emphasis on patient education regarding the safety profile and associated risks compared to the gained benefits.

## Background

Immune thrombocytopenia (ITP) is a hematological disorder in which there is an isolated decrease in number of circulating platelets [1]. In either primary ITP or secondary ITP, it is thought to be an immune mediated process [2, 3]. The disease may manifest from simple bruising to overt bleeding with an incidence in adults ranging from approximately 1.6 to 3.9 per 100,000 persons per year with a higher incidence in women than men [4–6]. Most adult patients presenting with acute ITP recover within weeks however some may progress to a chronic form of the disease [3].

Bee Venom therapy (BVT) is a well-known form of alternative and complementary medicine. There is a belief that ancient Egypt, Greece and China had applied BVT for patients suffering from rheumatism [7]. It is still being practiced in the Middle East as well as other parts in Asia. BVT is where acupuncture points are used to inject bee venom into the body either by direct sting or diluted injection [7].

There is no significant evidence supporting the effectiveness of BVT, but several trials have been and are currently conducted to support the use of BVT. There have been many reported complications of bee stings in the literature. These are mainly thought to be induced by the active components of bee venom. We may group these complications according to system affection; hematological, renal, liver, cardiovascular, musculoskeletal, central nervous system and multi-organ failure [8–16]. The most striking of these are potential anaphylactic reactions which may be life threatening [7]. These complications include three case reports of thrombocytopenia. In this report we describe a case of adult onset ITP following BVT without evidence of a known secondary cause of ITP. We aim to raise awareness of a possible complication of BVT.

## Case presentation

**Fig. 1:**
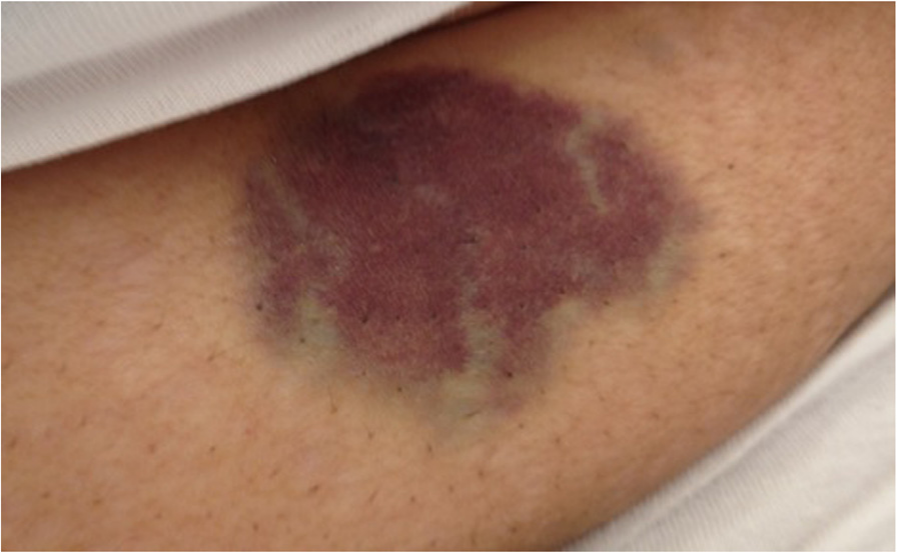
Abdominal ecchymosis size (5 cm diameter)

**Fig. 2:**
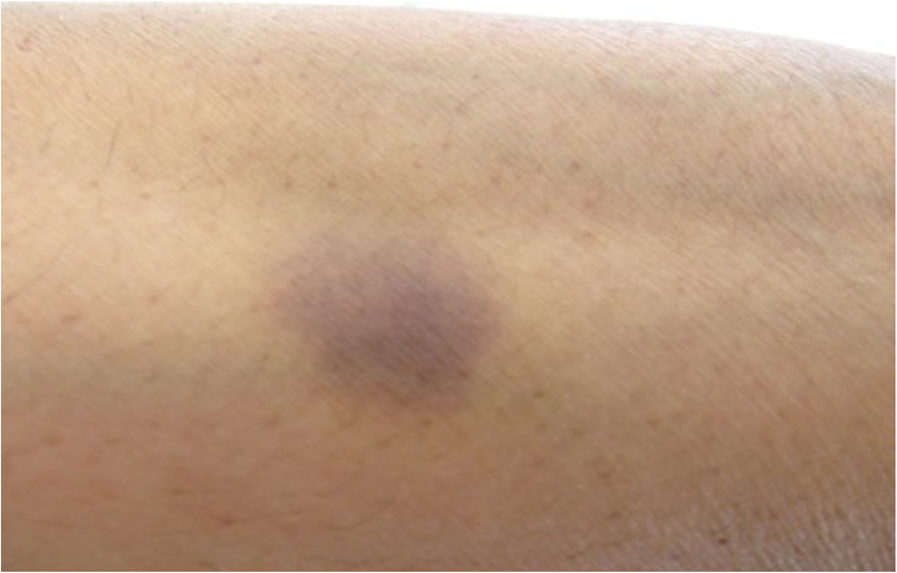
Right forearm ecchymosis size (2 cm diameter)

A 61 year old lady presented to the emergency department with 1 day history of bleeding from her gums. She denied any history of hematemesis, hemoptysis or epistaxis. Furthermore, she did not have melena or hematuria with no preceding history of flu-like symptoms. She noticed a non-itchy well demarcated rash, bluish in color on her right forearm and abdomen (Figs. 1 and 2).

With regards to her previous medical history, she was diagnosed with lumbar disc prolapse. Recently, she has started BVT sessions to relieve her pain. She received four once weekly direct bee sting sessions, first was a month prior to her presentation. Nevertheless, she is morbidly obese with a BMI of more than 40 and has bilateral knee osteoarthritis as a consequence. She did not use any new herbal or prescribed medications.

Upon arrival to the emergency department, she was fully oriented. She did not show any sign of hemodynamic instability. Systemic examination was unremarkable except for the skin, which showed two ecchymotic rashes. The first one was inferior to the umbilicus, round and macular, bluish in color, with a diameter of 5 cm (Fig. 1). The second was in the planter aspect of the right forearm distally; with a diameter of 2 cm (Fig. 2).

Laboratory investigations were as follows: Full blood count of WBC 5.8 × 10^9^/L, Platelets 9 × 10^9^/L (manual count: 15 × 10^9^/L), Hb 140 g/L. Her urea and electrolyte; Urea 3.3 mmol/L, Crea 41 mmol/L, K 3.5 mmol/L, Na 140 mmol/L, Glu 5 mmol/L, HCO_3_ 25 mmol/L. Liver function test and liver enzymes; Alkaline phosphatase 154 IU/L, ALT 41 IU/L, AST35 IU/L, Bil 11 umol/L, INR 0.94, APTT 32 s, protein 78 g/L, albumin 35 g/L, TSH 2 mmol/L. After reviewing her previous blood work up, she had a platelet count of 240 × 10^9^/L few weeks before starting her first BVT session.

Her peripheral blood smear showed: WBC; few activated lymphocyte and neutrophils with left shift, RBC; normocytic and normochromic, Platelets; giant platelets seen. Disseminated intravascular coagulation was ruled out based on laboratory test and blood film. She was assessed by a consultant hematologist and a diagnosis of ITP was established. Ultrasound abdomen showed a complete normal study. HCV, HBV and HIV screening was negative.

She was admitted to the hospital for 3 days. Initially, she received a 2 days course of immunoglobulin (IVIG 90 g once daily). After that she received a course of prednisolone (90 mg once daily for 2 weeks). She was clinically stable throughout her hospital stay with no further symptoms. On the day of discharge, her platelet count was 42 × 10^9^/L. The patient was followed up in the outpatient clinic after 8 weeks. She was asymptomatic and her platelet count was 162 × 10^9^/L.

## Conclusions

It is believed that BVT is effective in treating various conditions including rheumatoid arthritis, bursitis, tendonitis, post-herpetic neuralgia, multiple scleroses, gout, burns and infection [7]. There are a number of studies addressing the potential effectiveness of BVT in treating cancer patients [7]. Current research shows a potential therapeutic use of BVT in treating low back pain. However, despite the promising nature of the research the Food and Drug Administration (FDA) has not yet approved it [7].

ITP may be primary when no cause is identified. It accounts for approximately 80 % of all ITP cases [17]. Secondary causes include autoimmune causes (SLE, Antiphospholipid syndrome, Evans syndrome), Infectious (Hepatitis C, HIV, H.pylori), neoplastic (Chronic lymphocytic leukemia, lymphomas) and other rare causes [17]. All common secondary causes of ITP were excluded in our case.

In a review of the literature three case reports of bee sting induced ITP were identified. The first was reported by Tanphaichitr in 1982 [18]. The second case was about a 9 year old female who developed ITP with a platelet count of 15 × 10^9^/L post accidental bee sting in the mouth. This second case was reported by Nam dev in 2009 [19]. The third case reported in 2011 by Akbayram, in which a 4 year old boy developed an ITP with a platelet count of 5 × 10^9^/L after a honeybee bite [20]. The drop in our patient’s platelet count was suggestive to be due to BVT.

The pharmacological basis behind BVT is not fully understood. However, there are suggestions that bee venom has a number of pharmacological actions including analgesic, anti-inflammatory, and anti-cancer actions through multiple mechanisms [7]. The active components of bee venom include apamin, phospholipase A2 (PLA2), melitin, mast cell degradation peptide and histamine [21]. These components have an immunological effect on the body [22]. A number of studies tried to explain the pathophysiology behind the complications associated with use of bee venom therapy. The PLA2 is known to have a hematological affect including coagulation abnormalities [23]. Yuan et al. reported that PLA2 from bee and wasp venom inhibits platelet aggregation through the formation of lysophosphatidylcholine [24]. This may help us speculate that bee venom induces a host of immunological reactions. However, further research is required to clarify these mechanisms.

In conclusion, with the present knowledge of bee venom components and toxicity it is reasonable to associate thrombocytopenia as a complication of BVT however its use should be further studied to avoid potential consequences. Further research is needed to postulate guidelines and protocol for BVT. In the meantime, monitoring of the practice of BVT should be made, with an emphasis on patient education regarding the safety profile and associated risks compared to the gained benefits.

### Consent

Written informed consent was obtained from the patient for publication of this case report and any accompanying images. A copy of the written consent is available for review by the Editor of this journal.

## Abbreviations

BVT: bee venom therapy
HBV: hepatitis B virus
HCV: hepatitis C virus
HIV: human immunodeficiency virus
ITP: immune thrombocytopenia
IVIG: intravenous immunoglobulin
PLA2: phospholipase A2
